# *Corynanthe pachyceras* K. Schum (Rubiaceae) boosts sexual behavior, improves sexual hormones/semen quality and, modulates oxidative stress in a rat model of age-induced hypogonadism: an experimental study

**DOI:** 10.1093/sexmed/qfaf033

**Published:** 2025-05-06

**Authors:** Aubrile Julie Ndomgang, Henderson Herris Karl Ngombeu Zeugang, Yannick Baudouin Tchatat Petnga, Georges Romeo Bonsou Fozin, Aimé Césaire Tetsatsi Momo, Modeste Wankeu-Nya, Esther Ngadjui, Pierre Watcho

**Affiliations:** Unit of Animal Physiology and Phytopharmacology, University of Dschang, P.O. BOX 67 Dschang, Cameroon; Unit of Animal Physiology and Phytopharmacology, University of Dschang, P.O. BOX 67 Dschang, Cameroon; Unit of Animal Physiology and Phytopharmacology, University of Dschang, P.O. BOX 67 Dschang, Cameroon; Unit of Animal Physiology and Phytopharmacology, University of Dschang, P.O. BOX 67 Dschang, Cameroon; Unit of Animal Physiology and Phytopharmacology, University of Dschang, P.O. BOX 67 Dschang, Cameroon; Faculty of Health Sciences, University of Bamenda, P.O. BOX 39 Bambili, Cameroon; Laboratory of Animal Biology and Physiology, Department of Animal Organisms Biology, University of Douala, P.O. BOX 2701 Douala, Cameroon; Unit of Animal Physiology and Phytopharmacology, University of Dschang, P.O. BOX 67 Dschang, Cameroon; Unit of Animal Physiology and Phytopharmacology, University of Dschang, P.O. BOX 67 Dschang, Cameroon

**Keywords:** *Corynanthe pachyceras*, hypogonadism, aphrodisiac, androgen, antioxidant, old rats

## Abstract

**Background:**

Hypogonadism refers to a condition where the body does not produce enough testosterone. It is an important cause of male sexual dysfunctions and infertility. *Corynanthe pachyceras* is a medicinal plant with a claimed powerful aphrodisiac potential.

**Aim:**

This study investigated the capacities of *C. pachyceras* in boosting the sexual performance of age-related hypogonadic rat.

**Methods:**

Thirty-six aged (17-19-months-old) male Wistar rats (n = 6/group) were randomly distributed into six groups and orally treated with either distilled water (10 mg/kg), Viagra (5 mg/kg), aqueous or ethanolic extracts of *C. pachyceras* (8 mg/kg or 50 mg/kg) within two weeks. The copulatory activity was tested on days 0, 7, and 14 using receptive females. Body and sex organ weights, sperm parameters, sex hormones, oxidative stress markers, and penile nitric oxide (NO) were measured.

**Outcomes:**

The main outcome of this work is the confirmed efficacy of *C. pachyceras* in improving the reproductive performance of age-related hypogonadic rats.

**Results:**

The copulatory activity of the untreated hypogonadic aged rats (distilled water group) was low and marked by a significant increase in the sexual motivation (latencies and post-ejaculatory interval) and a decrease in the sexual performance (frequencies). Malondialdehyde level was increased, whereas the activities of superoxide dismutase and glutathione were decreased. Treatment of aged rats with the aqueous or ethanolic extracts of *C. pachyceras* brought important changes, shown by the significant decrease in the motivation parameters and the increase in the ejaculation frequency. Sperm count and motility (*P* < .01-.001), LH (*P* < .01), FSH (*P* < .01-.001), testosterone (*P* < .01), and NO (*P* < .05) levels that were altered in the untreated aged rats were increased in those receiving *C. pachyceras* extracts. The plant extracts also significantly (*P* < .05-.01) improved the antioxidant status by reducing the level of MDA and increasing that of SOD (*P* < .05–.01) and glutathione.

**Clinical Translation:**

*C. pachyceras* may serve as a potential therapeutic agent for improving the sexual health of androgen-deficient subjects such as aged men.

**Strengths and Limitations:**

*C. pachyceras* has androgenic, aphrodisiac, and antioxidant properties. However, its molecular mechanism is not yet determined. A well-structured clinical study needs also to be carried to confirm the effects of *C. pachyceras* on humans.

**Conclusion:**

Present findings showed that *C. pachyceras* can boost the reproductive conditions of age-induced hypogonadism rats through its androgenic, aphrodisiac, and antioxidant potentials. However, further studies are still needed to gain more medical insight into this plant.

## Introduction

Hypogonadism is a condition where the body does not produce enough testosterone. It significantly impacts health and fertility.^1^ For instance, spermatogenesis is dramatically reduced, leading to decreased fertility.^2^ This condition is associated with a decrease in serum testosterone of approximately 1% per year after the age of 30.^3^ It can be caused by many factors, including Klinefelter’s syndrome, obesity, genetics, oxidative stress, orchitis, pituitary and hypothalamic dysfunction, and aging.^4^ Hypogonadism can lead to the production of ROS or downregulated availability of antioxidants, leading to oxidative stress.^3^ This stress has been confirmed to be among the underlying mechanisms that induce harm to Leydig cells through triggering lipid peroxidation, inducing apoptosis, damaging mitochondrial activity, and reducing testosterone production.^3^ Hypogonadism alters sexual, physical, and mental functions. Sexually related manifestations include reduced libido, erectile dysfunction (ED), and ejaculatory dysfunctions. While a loss of sexuality could be considered normal and inevitable with the aging process, sexuality remains a key characteristic of masculinity.

Erectile dysfunction, the repeated inability to achieve or maintain penile erection for satisfactory sexual activity, is one of the most common disorders in men.^5^ The prevalence and severity of ED increase with age.^6^ The NOS/cGMP pathway has been deemed critical for erectile function.^6^ Basically, for a normal erection, penis must have an adequate testosterone concentration and blood pressure leading to the smooth muscle relaxation and penile vasodilation.^7^ Nitric oxide (NO) is a neurotransmitter that plays a key role in penile erection; it leads to the initiation of the synthesis of cyclic guanosine monophosphate and is involved in the decrease of calcium levels.^8^ However, this process depends on a complex balance and coordination of neurogenic, vascular, and humoral events,^9^ such as the sympathetic, parasympathetic, and nitrergic nerves, neurotransmitters, blood vessels, and cavernous muscles.^8^

Because ED may be associated with testosterone deficiency, androgen replacement is one of the important treatments that can alleviate hypogonadism symptoms by enhancing libido and sexual function.^8^ However, long-term testosterone treatment is uncertain, and prostate cancer is an absolute contraindication.^10^ Moreover, EDs are often treated with penile implant surgery, hormone therapies, or the use of phosphodiesterase type 5 inhibitors such as sildenafil citrate (Viagra) or tadalafil.^7^^,^^9^ These treatment options are expensive and constitute a source of harmful side effects.^11^ The effectiveness of some medicinal plants in the management of sexual dysfunctions has been reported. For instance, *Ficus asperifolia,*^12^  *Anogeissus leiocarpus,*^11^  *Camelia sinensis*, *Panax ginseng,*^13^ and *Eurycoma longifolia,*^14^ improve serum testosterone levels, sexual behavior, and performance.

*Corynanthe pachyceras* (Rubiaceae) is a lower-story forest tree growing in Sub-Sahara Africa. It is used in the Center Region of Cameroon for sexual vitality and libido enhancement or against EDs and genito-urinary infections. However, no scientific investigations have been made regarding its folkloric use. Previous studies have shown that this plant is less toxic,^15^ effective in the treatment of boils, chronic wounds, and possesses anti-infective potential.^16^

In our quest to enhance the value of medicinal plants, and considering the documented activities on *C. pachyceras*, this study was carried out to provide scientific arguments in favor of the aphrodisiac properties claimed by traditional practitioners. We used an experimental animal model that reproduces the main features of human androgen deficiency: the senescent rat model. To achieve this, we asked whether *C. pachyceras* possesses pro-sexual effects on a model of age-induced hypogonadism. We therefore hypothesized that the sexual stimulant effects of *C. pachyceras* could be mediated through improvement of sexual behavior, sex hormone biosynthesis, sperm parameters, and oxidative stress.

## Materials and methods

### Plant collection and extract preparations

Fresh barks from the trunk of *C. pachyceras* K. Schum (Rubiaceae) were harvested in May 2020 at Ngoumou in the Mefou-Akono Division, Center Region of Cameroon.

The aqueous extract (AE) of *C. pachyceras* was prepared by macerating 250 g of powder in 1000 mL of distilled water for 72 hours with occasional stirring. After filtration using a fine cotton thread and Whatman paper N°4, the filtrate was oven-dried (40 °C) to obtain 21 g of AE, giving an extraction yield of 8.4%.

The ethanolic extract (EE) was obtained after maceration of 250 g of *C. pachyceras* powder in 1000 mL ethanol for 72 hours at room temperature. After filtration, the filtrate was evaporated using a rotary evaporator (75 °C) under reduced pressure and 21.49 gr of EE were obtained (8.6%).

### Dose selection

The Human Equivalent Dose (HED), also referred to as the therapeutic dose, was found to be 8 mg/kg and was used in this study. The second dose, 50 mg/kg, was calculated using the formula of Nair and Jacobs^17^ which elucidates the conversion of doses between humans and animals.

### Animals

Adult females (5-6-month-old) and senescent male rats (17-19-month-old) were obtained from the animal house of the Faculty of Science, University of Dschang, Cameroon. They were maintained in a standard environment (22-25 °C; approximately 12 hours of light and 12 hours of dark cycle) and had food and water ad libitum. Adult females were used for behavioral test. All protocols in this animal experiment were conducted in strict accordance with the animal care and use guidelines of the scientific committee of the Department of Animal Biology, which follows the internationally accepted standard ethical guidelines for laboratory animal use and care as described in the European Economic Community guidelines, EEC. 2010 Council Directive 2010/63/EU of November 22, 2010.

### Preliminary phytochemical screening

Classes of metabolites present in the aqueous and EEs of *C. pachyceras* were identified using standard qualitative phytochemical screening methods as described by Harborne.^18^ Colorimetric tests and/or precipitate formation were performed for the presence of tannins, alkaloids, phenols, saponins, flavonoids, sterols, triterpenoids, anthocyanins, and anthraquinones.

### Animal treatment

Thirty-six aged male rats (17-19 months) were randomly distributed into six groups (n = 6) and orally treated. Control groups received distilled water (10 mL/kg/day) or Viagra (5 mg/kg/day). Experimental groups received aqueous or EE of *C. pachyceras* (8 or 50 mg/kg/day) within two weeks.

### Sexual behavior study

This test was conducted following the procedure described by Watcho et al.^12^ Briefly, each male was introduced into the mating cage. Five minutes after acclimatization, a receptive female was introduced into the cage and copulatory parameters were recorded during one hour, on days 0, 7, and 14 of treatment in a very low-light enclosure: The following sexual behavior parameters were analyzed: mount latency (ML, time from the introduction of the female till the first mount by the male); intromission latency (IL, the time taken from the introduction of the female to the first mount and vaginal penetration); ejaculation latency (EL, the time interval between the first intromission and ejaculation); mount frequency (MF, the number of mounts within a given time frame); ejaculation frequency (EF, the number of ejaculations from the time of introduction of the female rats to the male within a given time frame), Post Ejaculatory Interval (PEI, the time taken from ejaculation until the next intromission).

### Blood collection and tissue preparation

Rats were sacrificed under diazepam/ketamine anesthesia, and blood samples were collected from the catheterization of an abdominal artery and centrifuged for 15 min at 3000 rpm. The plasma was there after being gently pipetted and kept in sealed tubes at −20°C prior to the measurement of sexual hormones and total cholesterol. Testes, epididymis, prostate, seminal vesicles, and penis were exposed and removed, washed in saline solution, dried, and weighed. Relative sex organ weights were calculated using the following formula: Relative sex organ weight = (absolute sex organ weight/body weight) × 100. The penile tissue was removed, washed in cold phosphate-buffered saline (PBS), weighed, rapidly frozen in liquid nitrogen, and stored at -20 °C refrigerator. The left epididymis was dilacerated for the assessment of sperm motility and sperm density. The left testis was crushed in mortar containing Tris buffer (PH = 7.4) to make 15% (g/mL) homogenate which was pipetted for storage at -20 °C before measurement of oxidative stress markers (MDA, SOD, and Glutathione [GSH]).

### Sperm density and motility

Immediately after sacrifice, the left epididymis cauda of each rat was minced and thoroughly mixed in 10 mL of warm (36 °C) 0.9% NaCl. 10 μL of this mixture were transferred to a Malassez hemocytometer and observed under a light microscope (OLYMPUS, X400). Motile and non-motile spermatozoa were counted in 10 fields, and the percentage of motile spermatozoa was calculated using the following formula:

Percentage of mobile spermatozoa (%) = (number of mobile spermatozoa/total number of counted spermatozoa) × 100.^18^

For sperm density, Ngoula et al. protocol was used.^19^ A 20-fold dilution was made by mixing the sperm suspension with 0.9% NaCl solution and the mixture was shacken gently. 10 μL of this mixture were transferred to a Malassez haemocytometer, observed under a light microscope (OLYMPUS, X400), and spermatozoa were counted in 10 fields.

### Assessment of plasma sex hormones and total cholesterol

Testosterone (T), follicle-stimulating hormone (FSH), and luteinizing hormone (LH) were measured in the plasma using ELISA kits supplied by Monobind Inc., according to the manufacturer's instructions. Plasmatic total cholesterol was determined using a standard colorimetric kit (CORMAY).

### Measurement of testicular oxidative stress markers

The effect of plant extracts on oxidative stress was obtained by measurement of antioxidant status. Lipid peroxidation in testis was assessed by measuring the level of malondialdehyde (MDA) according to the method of Wilbur et al.^20^ Superoxide dismutase (SOD) was assessed according to the method of Misra and Fridovich.^21^ GSH content described by Giustarini et al. was used.^22^

### Measurement of penile nitric oxide (NO) level

Penile NO content was measured in penis homogenates using a {Citation}commercial measurement kit (Roche Diagnostics, Germany) based on colorimetric analyses at 540 nm.

### Testis histological analysis

Immediately after sacrifice, rat testes were fixed in 10% formaldehyde. Fixed material was washed out for 3-5 minutes in running tap water to remove the excessive fixative solution. Pieces of testes were then passed through the alcohol series for dehydration procedure, and tissues were embedded in paraffin and cut into 5 μm thick sections using a rotary microtome. Slides were hematoxylin–eosin-stained before examination of the architecture and diameter of seminiferous tubules using a light microscope (OLYMPUS, 400X).

### Statistical analysis

The normal distribution of data was tested using the Kolmogorov–Smirnov test. Data were presented as mean ± SEM (standard error of the mean). Weight and biochemical parameters were analyzed using one-way ANOVA, followed by a Turkey HSD post-test to separate means. All statistical comparisons for behavioral tests were done by ANOVA repeated measures, followed by a Bonferroni post-test. Statistical analysis was performed using GraphPad Prism software, Version 8.4.2. Values of *P* < .05 were considered significant.

## Results

### Phytochemical evaluation

Qualitative phytochemical screening of aqueous and EE of *C. pachyceras* revealed the presence of tannins, alkaloids, phenols, saponins, flavonoids, triterpenoids, anthocyanins, and anthraquinones.

### Effects of *C. Pachyceras* on the sexual behavior of aged rats

#### Effects on mount latency and frequency

The effects of treatments on ML and MF are shown in Table 1. A significant increase in ML was observed on day 7 without any change in MF in the untreated aged group during the treatment period. Treatments with *C. pachyceras* extracts or Viagra significantly decreased ML (*P* < .001) and increased MF (*P* < .001; day 14) in comparison to the untreated aged group and their respective initial data (Day 0). These reductions were more important for ML at the dose of 50 mg/kg of the EE of *C. pachyceras* on day 7 (*P* < .001 and *P* < .001, respectively, compared to the untreated aged group and day 0). Also, males treated with the EE (8 mg/kg) showed the highest increase on day 14 (*P* < .001 and *P* < .01, respectively, compared to an untreated aged group and day 0).

**Table 1:** Effects of *Corynanthe pachyceras* on mount [latency] and (frequency).

**Treatments**	**Day 0**	**Day 7**	**Day 14**
**Aged untreated (10 mL/kg DW)**	[146.33 ± 17.88]	[283.5 ± 9.89^a^]	[255.50 ± 17.21]
	(23.17 ± 1.70)	(8.67 ± 0.76)	(19.83 ± 3.62)
**Aged + Viagra**	[236.67 ± 9.43]	[23.67 ± 1.43^c^^d^]	[41.17 ± 4.80^c^^e^]
	(7.33 ± 1.78)	(17 ± 1)	(51.67 ± 4.45^c^^e^)
**Aged + AE 8**	[102.67 ± 4.39]	[50.33 ± 11.42^d^]	[63.17 ± 15.38^e^]
	(13 ± 1.18)	(18.17 ± 2.75)	(51.67 ± 4.58^c^)
**Aged + AE 50**	[233.17 ± 17.21]	[53 ± 3.02^c^^d^]	[20.83 ± 3.51^c^^e^]
	(8.67 ± 1.48)	(21.83 ± 0.54)	(50.17 ± 6.42^c^^e^)
**Aged + EE 8**	[188.33 ± 28.94]	[86.33 ± 11.80^d^]	[60 ± 7.14^b^^e^]
	(11 ± 0.82)	(21.83 ± 2.26)	(54 ± 4.61^b^^e^)
**Aged + EE 50**	[219.67 ± 22.35]	[35.33 ± 6.15^c^^d^]	[60.67 ± 5.87^c^^e^]
	(11.67 ± 1.71)	14.83 ± 1.51)	(41 ± 7.34^c^^e^)

N = 6. Values ​​are means ± SEM (standard error of the mean).

^a^, *P* < .05.

^b^, *P* < .01.

^c^, *P* < .001 compared to initial data (D0).

^d^, *P* < .001 compared to the group receiving distilled water on day 7.

^e^, *P* < .001 compared to the groups receiving distilled water on day 14.

The values in the brackets represent the latencies in seconds, those in the parentheses represent the frequencies. Viagra 5 mg/kg. AE 8 Aqueous extract (8 mg/kg). AE 50, aqueous extract (50 mg/kg). EE 8, ethanolic extract (8 mg/kg). EE 50, ethanolic extract (50 mg/kg).

#### Effects on intromission

According to Table 2, a significant increase was noted in the IL of the untreated aged animals on days 7 (*P* < .01) and 14 (*P* < .001) when compared to D0 data. On the contrary, Viagra and *C. pachyceras*-treated rats significantly (*P* < .001) decreased IL at the same time periods (7 days, 50 mg/kg of each extract; 14 days, all doses).

**Table 2:** Effects of *Corynanthe pachyceras* on intromission [latency] and (frequency).

**Treatments**	**Day 0**	**Day 7**	**Day 14**
**Aged untreated**	[150.50 ± 17.93]	[303.33 ± 12.57^a^]	[313 ± 22.30^b^^e^]
	(18.17 ± 2.50)	(7.33 ± 0.61)	(16.50 ± 3.46)
**Aged + Viagra**	[252 ± 14.57]	[24 ± 1.44^b^^c^]	[44.50 ± 5.12^b^^e^]
	(7 ± 1.81)	(15.17 ± 1.30)	(42.50 ± 6.49^b^^e^)
**Aged + AE 8**	[104.83 ± 4.13]	[51.17 ± 11.42^c^]	[63.83 ± 15.32^e^]
	(11.17 ± 1.66)	(13.67 ± B2.51)	(43.33 ± 7.01^b^^e^)
**Aged + AE 50**	[235.17 ± 17.27]	[64.33 ± 8.65^b^^c^]	[19.67 ± 3.12^b^^e^]
	(7 ± 1.57)	(17.67 ± 2.19)	(43.50 ± 6.30^b^^e^)
**Aged + EE 8**	[194.33 ± 29]	[87 ± 11.70^c^]	[54.17 ± 8.96^a^^e^]
	(10.17 ± 1.11)	(16.17 ± 3.53)	(44 ± 5.48^b^^e^)
**Aged + EE 50**	[221 ± 22.46]	[40.67 ± 6.86^b^^c^]	[64.33 ± 6.27^b^^e^]
	(12.67 ± 3.71)	(14.50 ± 2.93)	(30 ± 7.74^b^^d^)

N = 6. Values are presented as means ± SEM (standard error of the mean).

^a^, *P* < .01.

^b^, *P* < .001 compared to initial data (D0).

^c^, *P* < .001 compared to the group receiving distilled water on day 7.

^d^, *P* < .05.

^e^, *P* < .001 compared to the groups receiving distilled water on day 14.

The values in the brackets represent the latencies in seconds. Those in the parentheses represent the frequencies. DW, distilled water (10 mL/kg). Viagra 5 mg/kg. AE 8 aqueous extract (8 mg/kg). AE 50, aqueous extract (50 mg/kg). EE 8, ethanolic extract (8 mg/kg). EE 50, ethanolic extract (50 mg/kg).

In animals orally treated with Viagra or plant extracts, there was a significant increase in IF with a more pronounced effect recorded on day 14 (*P* < .001) (Table 2).

#### Effects on ejaculation latency and frequency

The EL remained unchanged within groups while a significant increase (*P* < .05-.001) of EF was observed in Viagra and *C. pachyceras-*treated rats on day 7 (Table 3).

**Table 3:** Effects of *Corynanthe pachyceras* on ejaculation [latency] and (frequency).

**Treatments**	**Day 0**	**Day 7**	**Day 14**
**Aged untreated**	[999.17 ± 158.85]	[1123.50 ± 111.76]	[956.33 ± 74.10]
	(2)	(1)	(1.67 ± 0.21)
**Aged + Viagra**	[534.33 ± 8.07]	[558.33 ± 5]	[804.17 ± 75.19]
	(1.67 ± 0.21)	(2^a^)	(1.5 ± 0.22)
**Aged + AE 8**	[1275.33 ± 196.21]	[872.67 ± 225.99]	[653.33 ± 94.66]
	(1.67 ± 0.21)	(2.17 ± 0.17^b^)	(2 ± 0.26)
**Aged + AE 50**	[703.17 ± 36.60]	[1025 ± 79.25]	[466.83 ± 124.01]
	(1.67 ± 0.21)	(2.33 ± 0.21^b^)	(2)
**Aged + EE 8**	[868.33 ± 172.38]	[786.33 ± 117.22]	[820.67 ± 156.32]
	(1.5 ± 0.22)	(1.83 ± 0.17^a^)	(1.67 ± 0.21)
**Aged + EE 50**	[1016 ± 67.23]	[506.67 ± 73.72]	[918.33 ± 91.65]
	(2 ± 0.45)	(1.83 ± 0.17^a^)	(2.17 ± 0.31)

N = 6. Values ​​are presented as means ± SEM (standard error of the mean).

^a^, *P* < .05.

^b^, *P* < .001 compared to the group receiving distilled water on day 7.

Values in brackets represent latencies in seconds. Those in parentheses represent frequencies. DW, untreated distilled water (10 mL/kg). Viagra 5 mg/kg. AE 8 aqueous extract (8 mg/kg). AE 50, aqueous extract (50 mg/kg). EE 8, ethanolic extract (8 mg/kg). EE 50, ethanolic extract (50 mg/kg).

**Table 4:** Effects of *Corynanthe pachyceras* on post-ejaculatory interval.

**Treatment**	**Day 0**	**Day 7**	**Day 14**
**Aged untreated**	578.17 ± 43.19	679 ± 23.61	559.17 ± 17.24
**Aged + Viagra**	290.67 ± 54.23	342.17 ± 63.76^c^	424.50 ± 97.34
**Aged + AE 8**	483.17 ± 69.73	436.67 ± 56.99	521 ± 34.67
**Aged + AE 50**	580.50 ± 17.15	349.83 ± 65.17^b^^c^	457.67 ± 86.43
**Aged + EE 8**	592.50 ± 79.90	372.17 ± 20.08^c^	396.67 ± 29.82
**Aged + EE 50**	492.83 ± 68.30	295.33 ± 38.68^a^^c^	422 ± 43.20

N = 6. Values are presented as means ± SEM (Standard error of the mean).

^a^, *P* < .05.

^b^, *P* < .01 compared to initial data (D0).

^c^, *P* < .001 compared to the group receiving distilled water on day 7.

Untreated DW, distilled water (10 mL/kg), Viagra 5 mg/kg. AE 8 aqueous extract (8 mg/kg). AE 50, aqueous extract (50 mg/kg). EE 8, ethanolic extract (8 mg/kg); EE 50, ethanolic extract (50 mg/kg).

#### Effects on post-ejaculatory interval

On day 7, only rats receiving plant extracts at the dose of 50 mg/kg showed a significant decrease (*P* < .05-.01) in the post-ejaculatory interval when compared to data from day 0. Animals receiving Viagra showed similar effects (Table 4).

### Effects of *C. Pachyceras* on body and sexual organ weights

**Figure 1:**
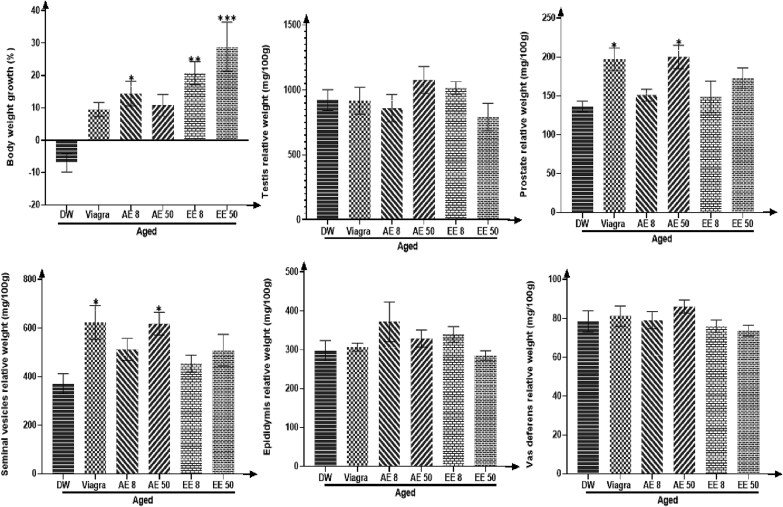
Effects of aqueous and ethanolic extracts of *Corynanthe pachyceras* on body weight variation and sexual relative organs weights of aged male rats after two weeks of treatment. N = 6. ^*^, *P* < .05 compared to DW. DW, untreated distilled water (10 mL/kg). Viagra 5 mg/kg. AE 8 aqueous extract (8 mg/kg). AE 50, aqueous extract (50 mg/kg). EE 8, ethanolic extract (8 mg/kg). EE 50, ethanolic extract (50 mg/kg).

Rats treated with *C. pachyceras* showed an increased body weight gain. This increase was significant (*P* < .05) in the groups receiving the AE at the dose of 8 mg/kg and the EE at all doses (*P* < .05-.01). Seminal vesicles and prostate weights were significantly (*P* < .05) increased in animals receiving Viagra and AE 50 mg/kg (Figure 1).

### Effects of *C. Pachyceras* on total cholesterol and sexual hormones (LH, FSH, and testosterone)

**Figure 2:**
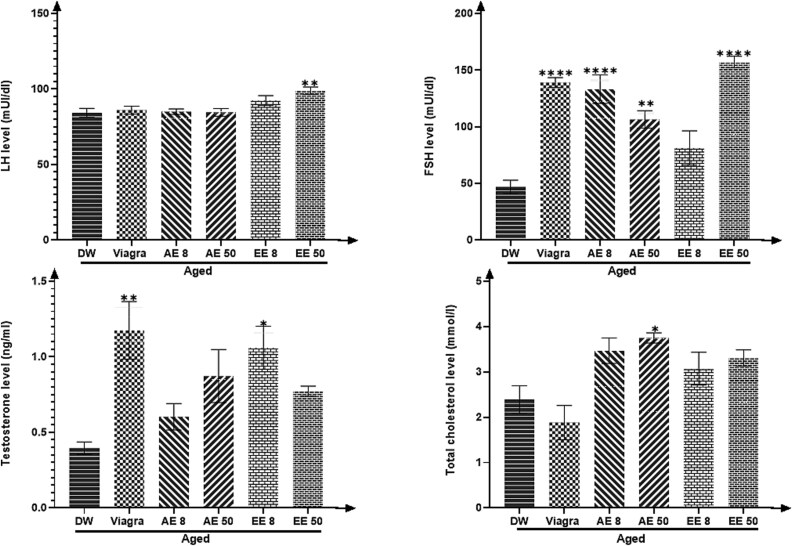
Effects of aqueous and ethanolic extract of *Corynanthe pachyceras* on LH. FSH, testosterone, and total cholesterol levels of aged male rats after two weeks of treatment. N = 6. ^*^, *P* < .05. ^**^, *P* < .01. ^****^, *P* < .0001 compared to DW. DW, untreated distilled water (10 mL/kg). Viagra 5 mg/kg. AE 8 aqueous extract (8 mg/kg). AE 50, aqueous extract (50 mg/kg). EE 8, ethanolic extract (8 mg/kg). EE 50, ethanolic extract (50 mg/kg).

*C. pachyceras* increased (*P* < .05-.0001) the plasma levels of total cholesterol (AE, 50 mg/kg), LH (EE, 50 mg/kg), FSH (except the EE 8 mg/kg group) and testosterone (EE, 8 mg/kg) when compared to the untreated aged animals. Viagra group almost produced similar effects (Figure 2).

### Effects of treatments on penile NO level

**Figure 3:**
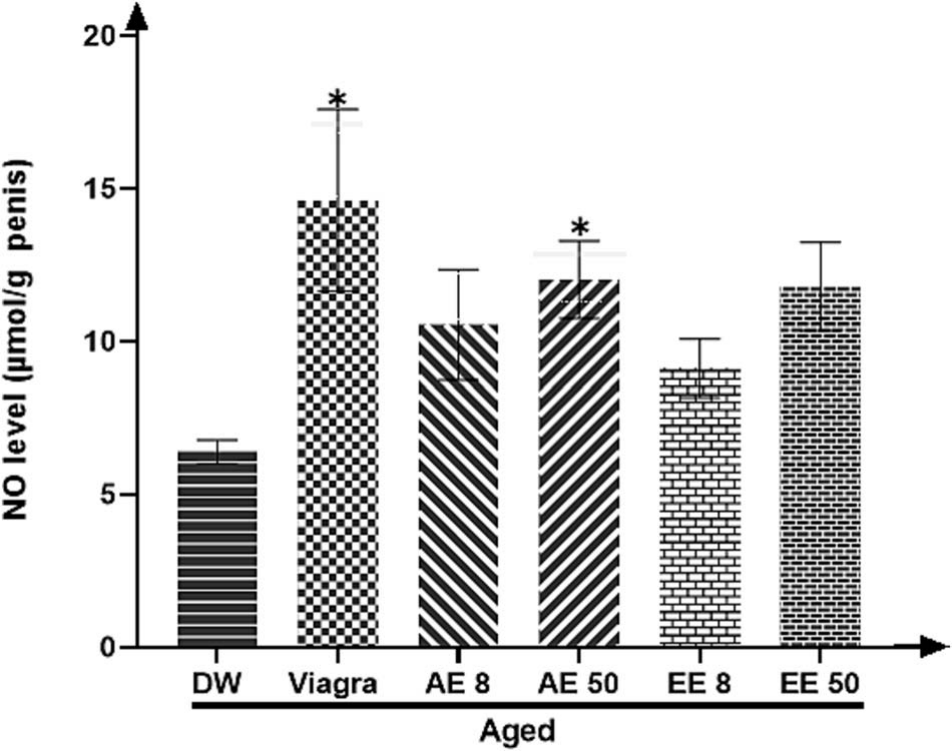
Effects of aqueous and ethanolic extract of *Corynanthe pachyceras* on NO level of aged male rats after two weeks of treatment. N = 6. ^*^, *P* < .05 compared to DW. DW, untreated distilled water (10 mL/kg). Viagra 5 mg/kg. AE 8 aqueous extract (8 mg/kg). AE 50, aqueous extract (50 mg/kg). EE 8, ethanolic extract (8 mg/kg). EE 50, ethanolic extract (50 mg/kg).

After treating the aged rats with either Viagra or the AE of *C. pachyceras* at the dose 50 mg/kg during two weeks, there was a significantly increase (*P* < .05) in penile NO concentration (Figure 3).

### Effect of *C. Pachyceras* on testicular oxidative stress markers

**Figure 4:**
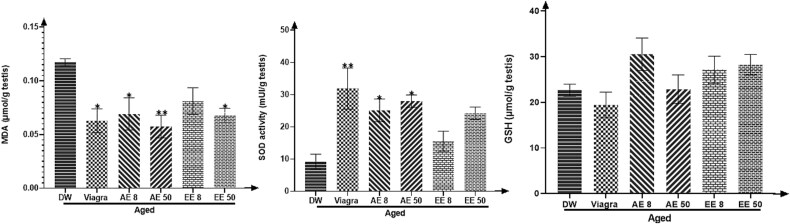
Effects of aqueous and ethanolic extract of *Corynanthe pachyceras* on MDA. SOD and GSH activities of aged male rats after two weeks of treatment. N = 6. ^*^, *P* < .05. ^**^, *P* < .01 compared to DW. DW, untreated distilled water (10 mL/kg). Viagra 5 mg/kg. AE 8 aqueous extract (8 mg/kg). AE 50, aqueous extract (50 mg/kg). EE 8, ethanolic extract (8 mg/kg). EE 50, ethanolic extract (50 mg/kg).

Treatment of aged rats with *C. pachyceras* extracts during two weeks was followed by a decrease in MDA concentration (*P* < .05-.01) (except the EE at dose 8 mg/kg) and an increase (*P* < .05-.01, AE) in SOD and GSH activities (Figure 4).

### Effects of *C. Pachyceras* on sperm density and motility

**Figure 5:**
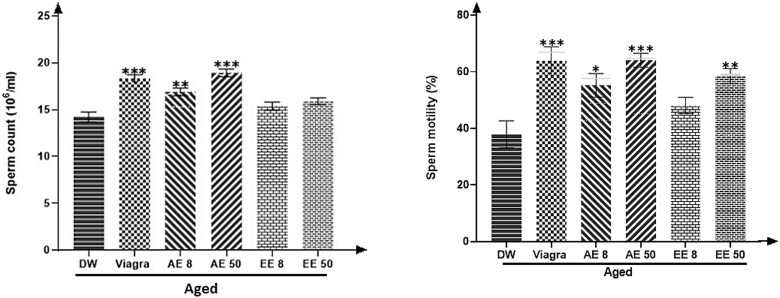
Effects of *Corynanthe pachyceras* on spermatozoa density and motility of aged male rats after two weeks of treatment. N = 6. ^**^, *P* < .01. ^***^, *P* < .001 compared to DW. DW: untreated distilled water (10 mL/kg). Viagra 5 mg/kg. AE 8 aqueous extract (8 mg/kg). AE 50, aqueous extract (50 mg/kg). EE 8, ethanolic extract (8 mg/kg). EE 50, ethanolic extract (50 mg/kg).

Figure 5 shows that both extracts of *C. pachyceras* significantly (*P* < .01-.001) improved the epididymal sperm motility of rats receiving the AE at both doses and sperm density at all doses except EE 8 mg/kg.

### Effects of *C. pachyceras* on testis histology

**Figure 6:**
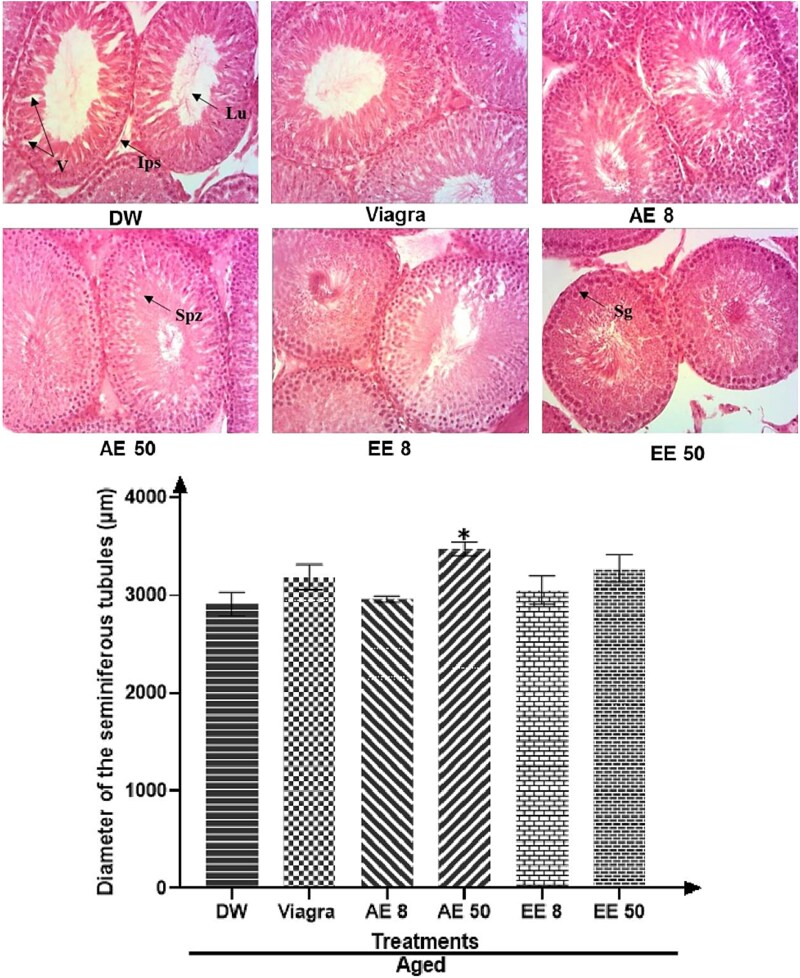
Effects of *Corynanthe pachyceras* on testis histology (hematoxylin and eosin, X 400) and diameter of seminiferous tubules of aged male rats after two weeks of treatment. Ips, interstitial space. Lu, lumen. Sg, spermatogonia. Spz, spermatozoa. V, vacuolation. N = 6. ^*^, *P* < .01 compared to DW. DW, untreated distilled water (10 mL/kg). Viagra 5 mg/kg. AE 8 aqueous extract (8 mg/kg). AE 50, aqueous extract (50 mg/kg). EE 8, ethanolic extract (8 mg/kg). EE 50, ethanolic extract (50 mg/kg).

Testes analysis of untreated aged rats showed an impaired architecture consisting of important degeneration, vacuolation, and irregular basement membrane with few spermatozoa in the lumen and reduced seminiferous tubules (Figure 6). However, *C. pachyceras* extracts markedly reversed the histopathological changes observed in the testes of an untreated aged rat.

## Discussion

This study examined the pro-sexual effects of aqueous and EEs of *C. pachyceras* in the aged male rats.

The copulatory behavior of laboratory rats is commonly used to evaluate the effects of synthetic and natural drugs on sexual function.^23^^,^^24^ In humans and animals, sexual behavior and sexual performance are generally reduced with the increase in age. Consequently, advanced age is considered a better condition for evaluating the effects of copulatory/androgen-like compounds.^25^ Since androgens play a key role in the initiation of sexual behavior, the drop observed in sexual behavior of the untreated aged rats may be due to the impairment of the hypothalamo-hypophysis axis, which further alters plasmatic testosterone. *C. pachyceras* significantly decreased the latencies and increased the frequencies of mount and intromission. This result suggests that *C. pachyceras* acts favorably in aged rats by stimulating sexual motivation and enhancing sexual performance leading to blood flow in the penis and, thus promoting erections followed by intromissions and ejaculations. This aphrodisiac effect of *C. pachyceras* may be assigned to the presence of alkaloids and saponins.^8^^,^^10^ Alkaloids are known to increase penile erection and to induce dilation of blood vessels in the sexual organs, while saponins act on the central nervous system and gonadal tissues, leading to enhancement of libido and copulatory performance through facilitation of penile erection.^8^^,^^10^ These overall activities induce vasodilatation and relaxation of the penile via a NO-dependent mechanism.^26^

Penile erection is initiated by NO from the non-adrenergic, non-cholinergic (NANC) nerves, and endothelial cells. NO then diffuses into the cavernosal smooth muscle and activates guanylate cyclase, resulting in accumulation of cGMP and subsequent activation of PKG, leading to the relaxation of cavernosal smooth muscle and penile erection.^26^ Moreover, the age-related decrease in androgen levels contributes significantly to the occurrence of ED, with the main cause being penile endothelial dysfunction due to a defect in NO production.^27^ In the present work, the level of NO in penile tissue of the animals treated with the plant extract was high compared to that of the control animals. This increase in penile NO level suggests that *C. pachyceras* extracts have the capacity to restore penile endothelial function of aged rats and, therefore, erectile function. These findings clearly support the fact that *C. pachyceras* may trigger penile erection and could therefore partially confirm its aphrodisiac potentials.

Male sexual behavior is highly dependent on testosterone, and its effect on copulatory enhancement is partially mediated through permissive actions on DA release in the medial preoptic area (MPOA).^28^ LH activates Leydig cells to produce testosterone, while FSH enhances Sertoli cells’ proliferation. Both hormones affect testosterone levels and sperm cell maturation. Moreover, FSH and testosterone impact the release and growth of spermatids. Thus, an appropriate concentration of FSH, LH, and testosterone is required for normal spermatogenesis. In the current study, we have found an increase in sperm density and sperm motility following the administration of *C. pachyceras*. All these effects may lead to enhanced fertility in males.^29^ Additionally, histological analysis of testes of the untreated aged animals presented deep structural and morphological alterations and a decrease in the diameter of the seminiferous tubules. Administration of *C. pachyceras* extracts reversed these spermatogenic and histological failures. These beneficial effects of *C. pachyceras* on spermatogenesis and testicular histology in aged rats could be due to its enhancing capacities on FSH and testosterone synthesis.

The growth and maintenance of sex organs are androgen-dependent processes.^30^ Any increase in plasmatic testosterone is generally associated with an increase in the secretory activity of the sexual organs. The evaluation of parameters such as sexual organs or body weight ratio, and cholesterol amount can give useful information regarding the androgenic effect of a substance. In this study, there was a significant increase in body, seminal vesicles, and prostate gland weights in the treated rats. This increase could be attributed to the androgenic properties of the plant, since it is well known that androgens, mainly testosterone, enhance the growth and activity of the accessory sex organs.^31^

Cholesterol is considered the absolute substrate for steroidogenesis.^32^ An increase in serum cholesterol may lead to a corresponding increase in testosterone level.^33^ The observed increase in cholesterol level could result from the effects of alkaloids present in the plant extracts.

Oxidative stress is known to be associated with homeostasis in many organs, such as testis.^34^ In this study, we found that the levels of MDA were decreased while SOD and GHS activities were increased in rats treated with *C. pachyceras* extracts compared with the untreated aged rats. These results suggest that *C. pachyceras* may protect against testicular dysfunction in aged rats by suppressing oxidative stress. These findings also suggest that the increase recorded in testosterone concentration may be due to the protective effects of *C. pachyceras* on the Leydig cells and seminiferous tubules.

Although the mechanisms of action explored in this study are crucial for controlling the effects of *C. pachyceras* in age-related hypogonadism rats, molecular approaches need to be used to ascertain the pathways involved. Also, given the quality of present results, a well-structured clinical study is also required to confirm the effects of *C. pachyceras* on humans prior to formal authorization and the use of this plant for hypogonadism management.

## Conclusion

Overall findings show that *C. pachyceras* possesses aphrodisiac and androgenic properties on aged male rats through improvement of sexual behavior and increase in sexual hormones (testosterone, LH, FSH) and NO levels. These findings could therefore serve as preliminary scientific data to support the folkloric use of *C. pachyceras*.

### CRediT taxonomy

A.J.N.: Conceptualization, Methodology, Project administration, Writing—original draft, Writing—review & editing. H.H.K.N.Z.: Investigation, Resources, Writing—review & editing. Y.B.T.P.: Investigation, Resources, Validation, Writing—review & editing. G.R.B.F.: Investigation, Resources, Validation, Writing—review & editing. A.C.T.M.: Investigation, Resources, Validation, Writing—review & editing. M.W.N.: Resources, Validation, Writing—review & editing. E.N.: Resources, Validation, Writing—review & editing. P.W.: Supervision, Writing—review & editing.
